# Brazilian Nurses Network Tackling the Antimicrobial Resistance (REBRAN): bringing the role of nurses from the shadow to the light

**DOI:** 10.1590/1980-220X-REEUSP-2023-0367en

**Published:** 2023-12-22

**Authors:** Lígia Maria Abraão, Rosely Moralez Figueiredo, Viviane Cristina de Lima Gusmão, Adriana Maria Félix, Caroline Lopes Ciofi-Silva, Maria Clara Padoveze

**Affiliations:** 1 United Health Group Brasil, Américas Serviços Médicos, Hospital Samaritano Higienópolis, São Paulo, SP, Brazil.; 2 Universidade de São Paulo, Escola de Enfermagem, São Paulo, SP, Brazil.; 3 Universidade Federal de São Carlos, São Carlos, SP, Brazil.; 4 Universidade Estadual de Campinas, Faculdade de Enfermagem, Campinas, SP, Brazil.

According to World Health Organization, the Antimicrobial Resistance (AMR) across humans and animals is one of the top ten threats to global health. The estimates suggest that by 2050, up to 10 million deaths could occur annually affecting the economies and directing even more people into situations of poverty^(1)^.

The cornerstone of the Global Plan for Antimicrobial Resistance is optimization of the antimicrobial use in a perspective of One Health^(2)^. Additionally, the Global Plan aims to improving awareness and understanding of AMR through effective strategies of communication, education, and training. One important strategy to contributes with this objective were the organization of the Global Annual Awareness Campaign, that was known as World Antimicrobial Awareness Week (WAAW), which happens every November, aiming at to raise global awareness and understanding on AMR^(3)^.

In line with the global strategies, Brazil has developed the National Action Plan for Prevention and Control of Antimicrobial Resistance in Brazil (PAN-BR), which targets mainly the rational use of antimicrobials^(4)^.

Reputed guidelines have strengthened the need for coordinated actions for the rational use of antimicrobials, such as the development of Antimicrobial Stewardship Programs (ASP). ASP is defined as “coordinated interventions designed to improve and measure the appropriate use of antibiotic agents by promoting the selection of the optimal antibiotic drug regimen, including dosing, duration of therapy, and route of administration”^(5)^. A multidisciplinary approach including physicians, pharmacists, microbiologists, intensivists, and nurses has been suggested as the best practice to be adopted^(6)^. However, for unknown reasons, nurses are still in the shadows and rarely appear in the literature regarding both AMR and ASP.

Staff nurses are identified as critical players in strategies to tackle AMR. Nurses represents the largest workforce in health services, with significant influence on care, and are frontline professionals engaged in the fight against AMR. This includes active participation in early detection of infection, specimen collection, antibiotic administration, monitoring treatment and adverse events and taking responsibility regarding antimicrobial treatment to optimize the use of antimicrobial agents. Nurses are uniquely positioned to deploy antimicrobial stewardship strategies and serve as a central hub for care integration which directly influence antimicrobial prescribing^(6,7,8,9)^. However, there are many challenges yet to face aiming at the full nurse’s awareness and engagement in the activities that will lead to keep AMR under control.

During the XVIII Brazilian Congress of Infection Control and Hospital Epidemiology in 2022, the Brazilian Nurses Network Tackling the Antimicrobial Resistance (REBRAN) was created. The proposal of REBRAN is to support and strengthen nurses in different health institutions to combat AMR, to acting effectively in ASP. The strategy involves the formation of a nurse group of Brazilian researchers and healthcare workers, with as partners of the civil society and representatives of health services, with the intent of building the necessary bridges between these different protagonists to promote research and educational activities in an integrated way. More broadly, the proposal is to establish a technical cooperation group of nurses on AMR, to encourage scientific discussions on the topics, development of research in the area, and contribution to the dissemination of knowledge and engagement of nurses to face this problem in Brazil.

REBRAN has bimonthly meetings, in which the members can discuss research and scientific AMR-related content, including mechanisms of AMR; antimicrobial modes of action; adverse reactions related to the use of antimicrobials; and strategies to AMR control. The network has built communication channels including email, WhatsApp group and social networks (Instagram e Twitter). Additionally, content on technical-scientific aspects has been periodically published on social media, to facilitate professionals’ access to updated scientific evidence on the topic. Also, network members have supported themselves awareness and training initiatives in relation to AMR carried out in health services.

Currently, the group has 207 members from all five Brazilian regions and different areas such as hospitals, outpatient settings, primary health care, education, just to mention a few.

On 27^th^ October 2023, REBRAN has achieved its first anniversary. REBRAN’s actions have been successful, as every meeting new member area added demonstrating the interest of nurses to be part of an initiative that can bring relevant contribution in the field. Through the participation in REBRAN, Brazilian nurses are raising the voices, leveraging their leadership, and bring up to the light the role of the nurses in the fight against AMR. We believe this initiative can be further a role model for other countries and inspire nurses worldwide.

## REBRAN ADRESSES

E-mail: rebran.contato@gmail.com; Instagram: https://www.instagram.com/rebran2022/; ex-Twitter: https://x.com/rebran_?s=11&t=4IYVDC2MHkQCG0XeKhd38Q
